# Remote fertility care: Are patients prepared to forego physician proximity in favor of accessibility?

**DOI:** 10.5935/1518-0557.20250172

**Published:** 2025

**Authors:** Maite del Collado, Daniela Paes de Almeida Ferreira Braga, Amanda Souza Setti, Sarthak Sawarkar, Santiago Munné, Edson Borges

**Affiliations:** 1 Fertility Medical Group/FertGroup, São Paulo - SP, Brazil; 2 Sapientiae Institute - Centro de Estudos e Pesquisa em Reprodução Humana Assistida, São Paulo - SP, Brazil; 3 Science For Everymind, São Paulo - SP, Brazil; 4 Sama Fertility, Princeton, NJ 08648, USA; 5 Progenesis, La Jolla, CA, USA; 6 Overture, Coral Gables, FL, USA; 7 Homu Health Ventures, Sant Andreu de Llavaneres, Spain

**Keywords:** telehealth, geographical barrier, assisted reproduction technologies

## Abstract

The prevalence of infertility is rising rapidly, affecting approximately one in every six individuals worldwide and impacting nearly 200 million people. Access to assisted reproductive technologies (ART) remains limited, as most countries fail to provide adequate ART services to those in need. Economic barriers are among the most significant obstacles to ART access. However, treatment costs rarely account for additional expenses such as transportation, accommodation, or lost work hours required for clinic visits, including consultations, monitoring exams, and procedures. These overlooked costs further increase the financial burden on patients. To mitigate geographical barriers, telehealth and remote monitoring technologies have emerged as promising solutions. Studies indicate that telehealth, when combined with remote ultrasound monitoring performed by local gynecologists, yields clinical outcomes comparable to traditional in-clinic monitoring, with high patient satisfaction. The development of remote ultrasound devices aims to enhance ART accessibility for patients facing logistical constraints. Research has demonstrated that these devices are non-inferior to conventional monitoring, providing similar oocyte and embryo outcomes while offering additional benefits such as increased patient autonomy, discretion, reduced stress, and lower costs. A survey of 146 Brazilian infertility patients assessed their acceptance of the remote ultrasound device, revealing that 88.3% were willing to conduct ultrasounds at home, provided that the image quality and diagnostic accuracy were comparable to those of clinic-based examinations. While infertility patients appear receptive to remote fertility care, concerns persist regarding the efficiency and reliability of remote monitoring. Therefore, identifying the most suitable patient profile for this approach is essential, with those already familiar with ART processes potentially being the most appropriate candidates.

The prevalence of infertility among individuals of reproductive age is increasing exponentially and is now estimated to affect one in every six individuals, impacting nearly 200 million people worldwide (WHO, 2023; Ombelet *et al.*, 2025). The right to access infertility treatment is a matter of human dignity, as affirmed in international statements by the United Nations (UN. Committee on Economic, 2000). The World Health Organization (WHO) acknowledges that the provision of high-quality family planning services, including fertility care, is a fundamental component of reproductive health (WHO, 2024). However, unlike most medical treatments, access to assisted reproductive technologies (ART) is not universally guaranteed for individuals diagnosed with infertility who seek medical assistance to achieve pregnancy. It was estimated that to reach a population who need for ART, at least 1500 couple per million inhabitants should have access to IVF per year (ESHRE Capri Workshop Group, 2001), and as we are aware, the majority of countries are far from achieving this number.

It is well recognized that various barriers hinder the attainment of the required number of patients for ART treatments, particularly in vitro fertilization (IVF). Among these, the most significant appears to be the economic barrier.

The average cost of IVF cycle among countries differs substantially. Evidence from a global study concluded that the total costs for a cycle with a fresh embryo transfer leading to a live birth could varied between € 4,108 - € 12,314 (Matorras *et al.*, 2023). Notably, these cost calculations for treatment rarely consider the potential economic impact associated with transportation, accommodation, or even lost work hours or days that certain patients may incur due to the physical distance to the fertility clinic. This additional cost due to the distance to the clinic may be a decisive factor when patients consider whether to undergo fertility treatment. Indeed, a work conducted by Lazzari *et al*. (2022) concluded that women who lived within 15 km of a fertility clinic were 21% more likely to undergo ART treatment than those who lived further than 60 km away. This may explain why the review by Mackay *et al*. (2023) identified geographical barriers as the most frequently reported obstacle to accessing assisted reproductive treatments across studies.

In addition to the first consultation, the oocyte retrieval and embryo transfer day, depending on the treatment protocol and the woman’s response to ovarian stimulation, the patient may need to visit the clinic for ultrasound monitoring of the stimulation progress on an additional 4 to 5 days, which can result in a significant additional expense within the treatment. In this scenario, in order to mitigate this geographical barrier, the telehealth, or the use of modern technology to bridge the gap, driven by the necessity during the COVID-19 pandemic, has provided us with valuable insights into a more patient-friendly approach to medicine.

Although telemedicine is widely documented in cardiology, endocrinology, dermatology, and general practice, there is limited reporting on its application in reproductive medicine. In reproductive health, it has been verified that patients who used telehealth, with ultrasound performed by a local gynecologist for cycle monitoring, reached the same total and mature oocyte numbers and equivalent clinical pregnancy and live birth rates as patients who underwent consultations and ultrasounds in the ART clinic, with 73% of patients being highly satisfied with telehealth (Mikhael *et al.*, 2021). In a recent meta-analysis, including almost 5,000 patients from eleven studies, similar clinical outcomes were found between conventional and telehealth monitoring, with higher levels of satisfaction reported for remote consultations (Tran *et al.*, 2024).

Some authors have raised the question that ultrasonographic monitoring of the cycle, being a relatively simple technique, may distance clinicians from more complex techniques that rely more on their expertise. In 2009, the remote ultrasound device or Self-Operated Endovaginal Telemonitoring (SOET), was conceived to enhance accessibility to assisted reproduction treatments for patients who, due to geographical or time limitations, are unable to attend a clinic for cycle monitoring (Gerris *et al.*, 2009). Since then, Gerris’s research group has been conducting studies to evaluate the efficacy of this device, demonstrating through a randomized controlled trial (RCT) that SOET is non-inferior to traditional monitoring with respect to the total and mature oocyte count, as well as the total and high-quality embryos collected (Gerris *et al.*, 2014). Furthermore, the patients reported higher contentment for both the patient and partner, a greater feeling of empowerment, more discretion, increased partner participation, and a trend towards less stress compared to conventional monitoring. As mentioned by the authors, a more suitable patient for home ultrasound is a woman who has undergone at least one cycle, is familiar with the appearance of growing follicles, faces logistical challenges in attending the clinic due to professional or domestic responsibilities or geographical location, is comfortable with basic vaginal manipulation, and is open to new approaches.

Brazil, an upper-middle-income country, is the fifth largest country in the world by land area, with 8.5 million km^2^. Due to its vast size, Brazil faces geographical barriers both between states and within each state. Moreover, in metropolitan cities like São Paulo, patients may take not just minutes, but hours to reach a fertility clinic. This physical and temporal distance not only incurs economic costs but also emotional costs for a patient already navigating a journey that can be stressful at many stages of the fertility treatment process.

To assess Brazilian patients’ receptiveness to this new approach, 146 infertility patients were surveyed regarding the use of this remote fertility device, the SOET. Among the respondents 57.5% were in their first ART cycle, and 76.71% were over the age of 35. When asked whether they would feel comfortable using an ultrasound device and performing a self-examination while receiving real-time instructions on how to maneuver the probe, 70.5% of participants responded affirmatively (Figure 1A). Additionally, 88.3% expressed interest in performing an ultrasound at home provided the image quality and report were comparable to those of a clinic-based examination (Figure 1B).

**Figure 1:**
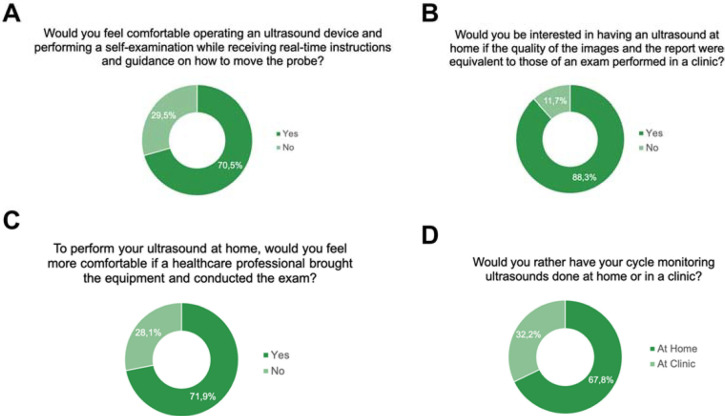
Patients’ perceptions of the implementation of remote fertility care for performing ultrasounds at home.

Although 67.8% of patients preferred undergoing ultrasound at home rather than at the clinic, 71.9% still preferred having a healthcare professional visit their home to perform the exam, suggesting understandable concerns about self-examinations (Figure 1C and D). When asked how much they would be willing to pay for this convenience, the majority responded that they would pay R$1,000 for this home ultrasound option. The primary reasons for choosing this option were transportation time (41.1%), 39.7% stated it was more convenient, and 9.6% mentioned the availability of a more flexible schedule. Our survey results provide insights into Brazilian patients’ perceptions of the possible implementation of a remote fertility care in a country with severe geographical barrier.

There is no doubt that remote devices, such as SOET, combined with telemedicine, could help improve access to ART for couples who choose not to undergo treatment due to the economic and emotional costs associated with traveling to the clinic multiple times throughout the treatment. However, as our survey suggested, despite a preference for home-based ultrasounds, patients still favor having a professional performing the procedure, reflecting ongoing concerns about the efficiency of remote devices. This is reflected in the findings of the latest meta-analysis, which concluded that, despite high satisfaction, telemedicine still raises concerns regarding its efficiency (Tran *et al.*, 2024).

Ultimately, patients may feel that part of the responsibility for the treatment falls on them due to their dependence on the proper use of the device. This matter requires further discussion among healthcare professionals, as well as between physicians and patients. Therefore, it may be worth reflecting on the patient profile that would be best suited for this approach, with those already familiar with the processes possibly being the most suitable candidates.

In conclusion, patients appear receptive to innovations that streamline the assisted reproduction journey. It may be appropriate to foster discussions on the advantages and limitations of these remote devices, as well as to identify the patients who could benefit most from their use, while ensuring that the responsibility associated with operating the equipment does not become a burden for them.
